# One-step photonic curing of screen-printed conductive Ni flake electrodes for use in flexible electronics

**DOI:** 10.1038/s41598-021-82961-3

**Published:** 2021-02-09

**Authors:** Bilge Nazli Altay, Vikram S. Turkani, Alexandra Pekarovicova, Paul D. Fleming, Massood Z. Atashbar, Martin Bolduc, Sylvain G. Cloutier

**Affiliations:** 1 Institute of Science and Technology, Marmara University, Istanbul, 34722 Turkey; 2 Chemical and Paper Engineering, Western Michigan University, Kalamazoo, MI 49008-5462 USA; 3 Electrical Engineering, École de Technologie Supérieure, 1100 Notre-Dame Ouest, Montréal, QC H3C 1K3 Canada; 4 Electrical and Computer Engineering, Western Michigan University, Kalamazoo, MI 49008-5462 USA; 5 Mechanical Engineering, Université du Québec À Trois-Rivières, 555 University Blvd, Drummondville, QC J2C 0R5 Canada

**Keywords:** Energy science and technology, Engineering, Materials science, Optics and photonics

## Abstract

Photonic curing has shown great promise in maintaining the integrity of flexible thin polymer substrates without structural degradation due to shrinkage, charring or decomposition during the sintering of printed functional ink films in milliseconds at high temperatures. In this paper, single-step photonic curing of screen-printed nickel (Ni) electrodes is reported for sensor, interconnector and printed electronics applications. Solid bleached sulphate paperboard (SBS) and polyethylene terephthalate polymer (PET) substrates are employed to investigate the electrical performance, ink transfer and ink spreading that directly affect the fabrication of homogeneous ink films. Ni flake ink is selected, particularly since its effects on sintering and rheology have not yet been examined. The viscosity of Ni flake ink yields shear-thinning behavior that is distinct from that of screen printing. The porous SBS substrate is allowed approximately 20% less ink usage. With one-step photonic curing, the electrodes on SBS and PET exhibited electrical performances of a minimum of 4 Ω/sq and 16 Ω/sq, respectively, at a pulse length of 1.6 ms, which is comparable to conventional thermal heating at 130 °C for 5 min. The results emphasize the suitability of Ni flake ink to fabricate electronic devices on flexible substrates by photonic curing.

## Introduction

Printing technologies and functional inks have been integrated to produce printed electronic devices and circuits on thin, lightweight and flexible materials in a timely and cost-effective manner^1–5^. The additive nature of printing that is free from acid etchants or wet processing chemicals assists in reducing the production steps and waste generation in comparison to conventional manufacturing for the fabrication of electronics^6–10^. In printed electronics, metallic inks containing precious or base elements can be deposited on papers, polymers, ceramics, metals or fabrics in a layer-by-layer manner by printing and then sintered to improve or bring out their electrical functionality^11,12^. The functionality of the printed ink layer is directly influenced by the complex interactions between the printing method; substrate characteristics, such as surface energy, wettability, topography, smoothness/roughness, absorbency; and ink characteristics, such as particle shape and size, rheology, surface tension and solid content^13^.

Ink spreading, drying mechanisms and the interface quality between the substrate and ink also affect the homogeneity of the printed ink and thickness^14^. Wettability is one of the quality parameters of substrates that occurs when an ink meets the surface of the substrate. The two intersect at an angle depending on the ink and substrate types, called the contact angle, which can be altered by surface treatments or the choice of materials to improve wettability^14^. A certain time is required for inks to wet substrates, called wetting time, which is strongly influenced by the flow properties of the inks, spreading of the ink drop and penetration into porous substrates through capillary flow mechanisms^15^. If the substrate is coated, laminated or saturated, penetration occurs by the diffusion mechanism^16,17^. Exploring the wettability mechanism significantly affects the decisions on the ink and substrate compatibility, printing method and postprinting process choices as well as profitability^18,19^.

Postprinting processes include ink drying and sintering steps that decompose the binder, an ink vehicle and additives that keep metal particles dispersed and stabilized in a formula^2, 20^. Conventional drying systems for metallic inks require processing times that range from minutes to hours at high temperatures and adversely affect heat-sensitive substrates with low glass-transition temperatures^21^. Photonic light irradiation has been reported to show great promise to sinter metallic inks at high temperatures in milliseconds while maintaining the integrity of flexible polymer and paper substrates without structural degradations such as caused by charring or decomposition^22–35^. The rapid high heat generated during photonic curing is dissipated via thermal conduction at the interface of the substrate using the thermal mass of the substrate^22^. In the process, neck formation phenomena occur between metallic particles and cause grain growth of particles; then, pores are removed, and a dense continuous conductive layer is achieved^36–39^.

Metallic particles for electronics inks have largely been limited to precious metals such as gold (Au), platinum (Pt) and silver (Ag) or base metals such as copper (Cu), which enable a wide range of applications, including sensors, solar panels, batteries, light sources and wearable electronics^40–44^. In recent years, Ni has evoked significant interest both in academia and industry for a wide variety of applications due to its high temperature coefficient of resistance (TCR) and sensitivity, electrical and thermal conductivity, resistance to oxidation-corrosion, mechanical strength and magnetic behavior that cannot be replaced by other common precious noble metals^45–52^. The applications of Ni vary from multilayer ceramic capacitors and interconnectors to optical antennas and magnetic sensors, switches and actuators^53^. Its TCR and sensitivity have shown great promise, especially in resistance temperature detector (RTD) sensor applications^54^. The metals used in RTD applications in general are Au, Pt and Ag and Cu. Among these, Pt is the most commonly used metal in RTD applications due to its high accuracy; however, it is expensive and suffers from a low response and TCR value^55,56^. Similarly, Au and Ag are expensive and have low TCR values^57,58^. The usage of Cu is limited since it is prone to oxidation at low temperatures^59^. Ni, on the other hand, has the highest sensitivity and TCR relative to the other metals^36^.

Ni particles are usually spherical in shape. However, different particle sizes, size distributions and morphologies can be obtained by changing the parameters of the chemical reaction process, such as temperature, reaction time and reactant concentration^60^. The shape of metal particles impacts the structural properties and performance of the functionally printed ink layer^61^. Therefore, the curing process requires optimization depending on the chemical composition of the particles along with their size, distribution, shape, and degree of agglomeration^62^. Altering the curing process by changing the temperature, time, or pressure (ambient vs. inert) can cause significant variation in the performance for the same material due to the change in grain boundaries and specific surface area of metal particles^36,63^. Moreover, when the active component of an ink differs from traditional powders, such as flake-shaped metal particles, it requires higher temperatures than spherical particles^61^. It is essential to carefully optimize the curing parameters in accordance with the material properties to achieve optimum functionality. The only study found on photonic curing of Ni film in the literature was based on spherical particles deposited via spin coating on a PI substrate by a two-step sintering process^64^. In this study, single-step photonic curing is reported for screen-printed Ni flake particles on SBS paperboard and PET film. The wettability of the substrates was also characterized to investigate the effect of porous and nonporous substrates on the electrical properties of Ni ink as well as ink transfer and the amount of ink usage.

## Experimental procedure

### Ink rheology and the structure of Ni particles and substrates

The rheology of the Ni flake ink paste prototype (*Metalon HPN-DEV 79–89-66; NovaCentrix: Austin, TX*) was assessed using an AR 200 dynamic stress rheometer (*TA Instrument: New Castle, DE*) in a 20 mm 2° cone-parallel plate geometry. The shear rate of the steady-state flow test was varied from 0.1 to 1000 s^−1^ at 23 °C. A temperature ramp test was conducted using a Peltier plate from 20 to 60 °C at a constant shear rate of 100 s^−1^. The composition of the ink reported by NovaCentrix was 50 to 90% (w/w) nickel, 2 to 15% diethylene glycol monobutyl ether, 0 to 20% isopropyl alcohol and a propriety binder. The solid content level was 62%.

The Ni flake ink sample was placed in ~ 25 ml of nanopure water and placed in a sonicator for 15 min to form a suspension. A glass stir bar was used to place a drop of the suspension on acetate film for drying. The particle shape was imaged using field emission scanning electron microscopy (FE-SEM) (*Fujifilm: Greenwood, SC*).

An FTA200 (*First Ten Angstrom; Portsmouth, VA*) video system with FTA32 software was employed to measure the wettability of substrates using the water sessile drop method under ambient conditions. The surface of substrates was characterized with a Contour GT-K vertical scanning 3D optical white light interferometer microscope (*Bruker Corp.: Billerica, MA*) by using the published conditions^65^ and with a MultiMode 8 Atomic Force Microscope (AFM) using a Nanoscope V Controller (*Bruker Corp.: Billerica, MA*). A total of 100 × 100 µm scans were acquired in contact mode, while 10 × 10 µm scans were acquired in tapping mode. 2D and 3D height images were plotted to show the relative roughness. The thickness of the substrates and Ni ink films was measured with a digital thickness gauge (*Technidyne: New Albany, IN*).

### Digital file creation and screen printing of Ni electrodes

**Figure 1:**
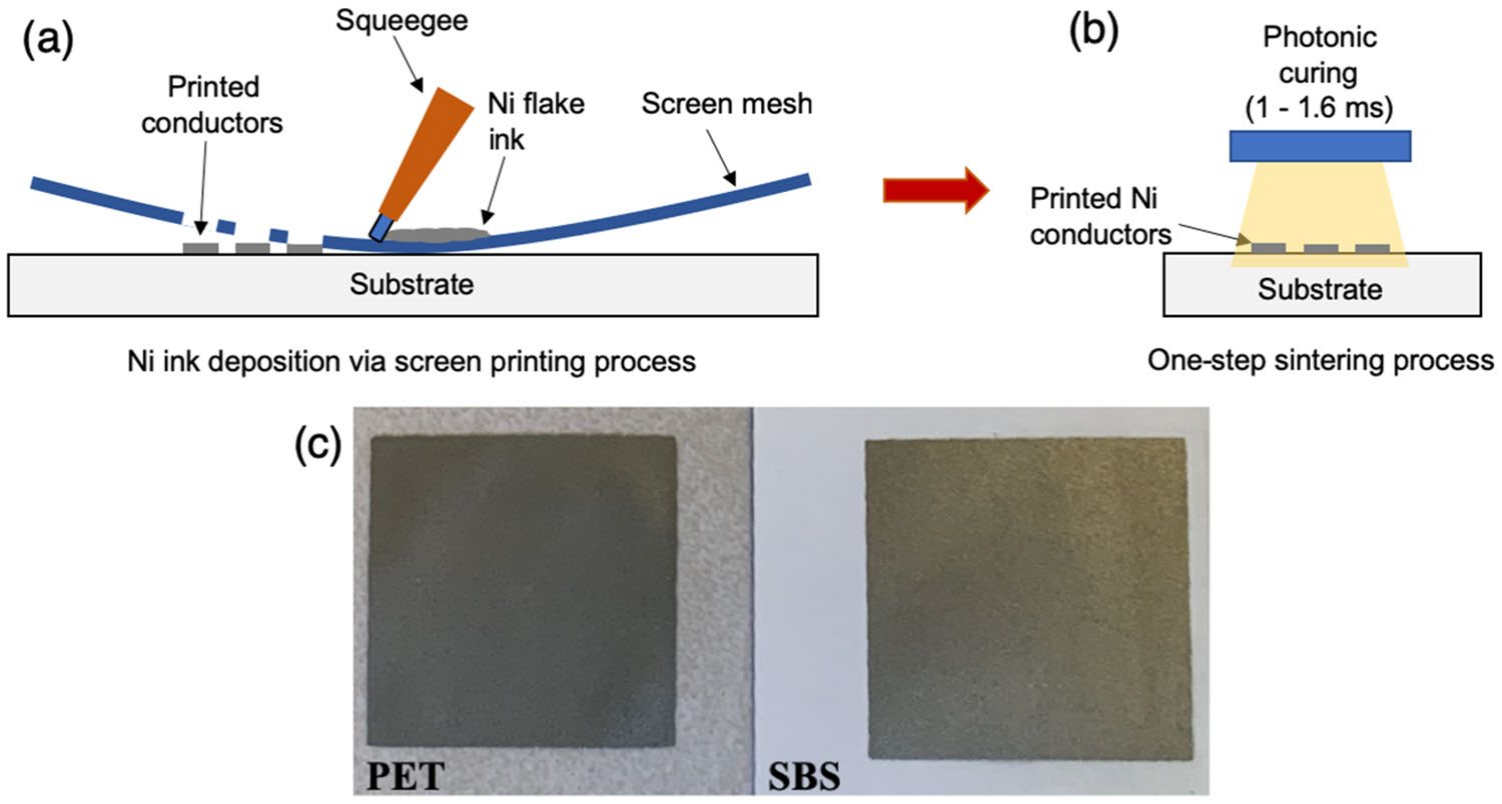
Schematics of electrode preparation: (**a**) screen printing, (**b**) photonic curing process and (**c**) sintered Ni on PET and SBS.

The pattern was designed in Adobe InDesign software with dimensions of 1.5 × 1.5 cm. A precut serigraphy emulsion (*Chromaline: Duluth, MN*) was applied on 165 threads per inch water wetted mesh and dried for 24 h. The plastic backing of the emulsion was peeled off, and the positive transparent film was placed on the emulsion and exposed to UV light for 1 min (*Lawson, St. Louis, MO*). Unexposed nonimaged areas were cleaned using pressurized water and dried for an hour at room temperature. Ni ink was printed on PET (*Melinex ST506; DuPont: Chester, VA*) and SBS (*C2S, 144-lb., 10 pt.; International Paper: Chicago, IL*) with a 45° angle squeegee stroke and sintered upon printing (Fig. 1). The substrates were weighed before and after printing using a Vibra CG electronic balance (*0.0001 g; Shinko Denshi Co. Ltd.: Tokyo, Japan*) to calculate the Ni ink usage.

### Photonic curing procedure

A PulseForge 1200 (*NovaCentrix: Austin, TX*) photonic curing equipment was employed to investigate the optimum curing settings for printed Ni ink on both substrates. A factorial experiment was designed by varying the pulse length from 1.0 to 1.6 ms in steps of 200 µs. The system was calibrated before the trials using its bolometer. The voltage, web speed and overlap settings were kept constant at 450 V (V), 20 feet/minute and 2 at once through mode, respectively. The overlap factor represents the average number of pulses that are received by the substrate^66^. The total exposure energy was varied between 4.8 and 6.8 J/cm^2^. Photonic light was generated from a flashlamp with wavelengths ranging from 200 to 1500 nm. The thermal profile of the curing conditions was simulated using the SimPulse software of the curing equipment.

### Printed Ni ink film characterization

Scanning electron microscopy (SEM) (EVO MA-15, *Carl Zeiss SMT Ltd.: Pleasanton, CA*) was used to image the printed Ni film surface, particle shape and microstructure. A 4-point probe source meter (*Model 2400; Keithley: Cleveland, OH*) was used to measure the electrical properties of the printed conductive patterns. The Haldor Topsøe geometrical factor method was used to calculate sheet resistance (Ω/sq) based on the printed line width and length^67,68^ for the volume resistivity calculations (Ωm). Transmission and reflectance of the printed Ni ink film was measured with a spectrometer that emits light in the UV and visible spectrum range (*DT2000, StellarNet, Inc.: Tampa, Florida*).

## Results and discussions

**Figure 2:**
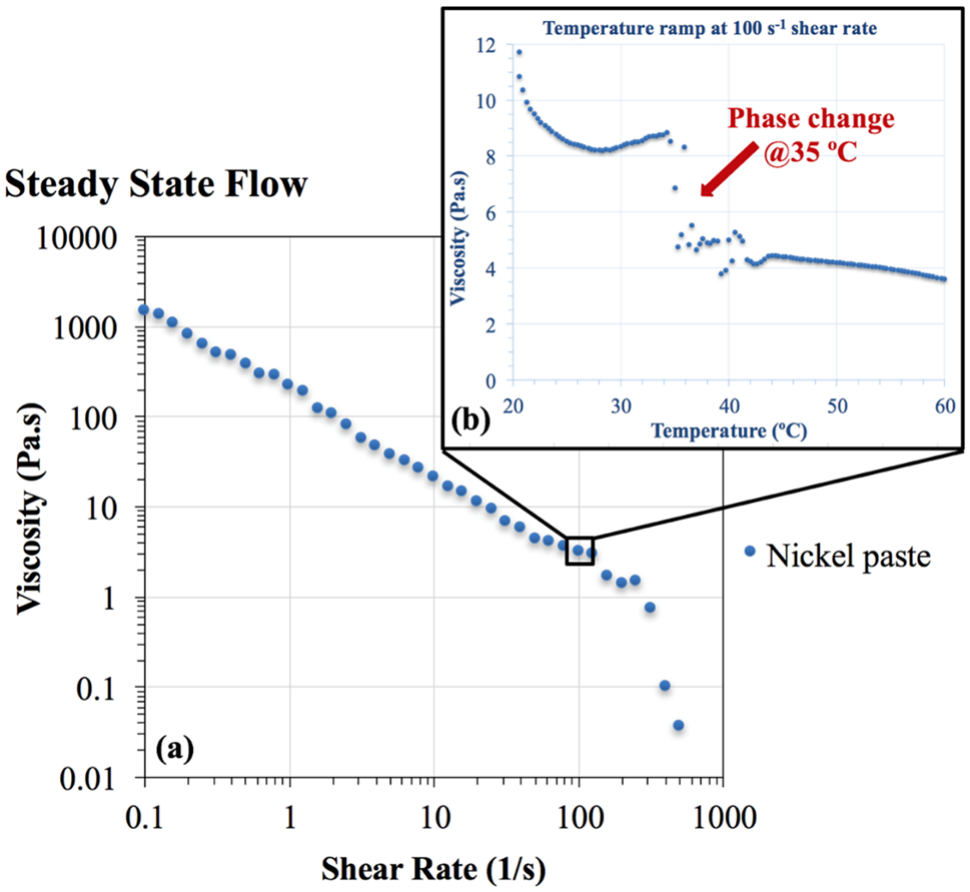
Ni ink flow curves: (**a**) viscosity dependence on the shear rate, (**b**) viscosity dependence on temperature measured with a constant 100 s^-1^ shear rate.

**Figure 3:**
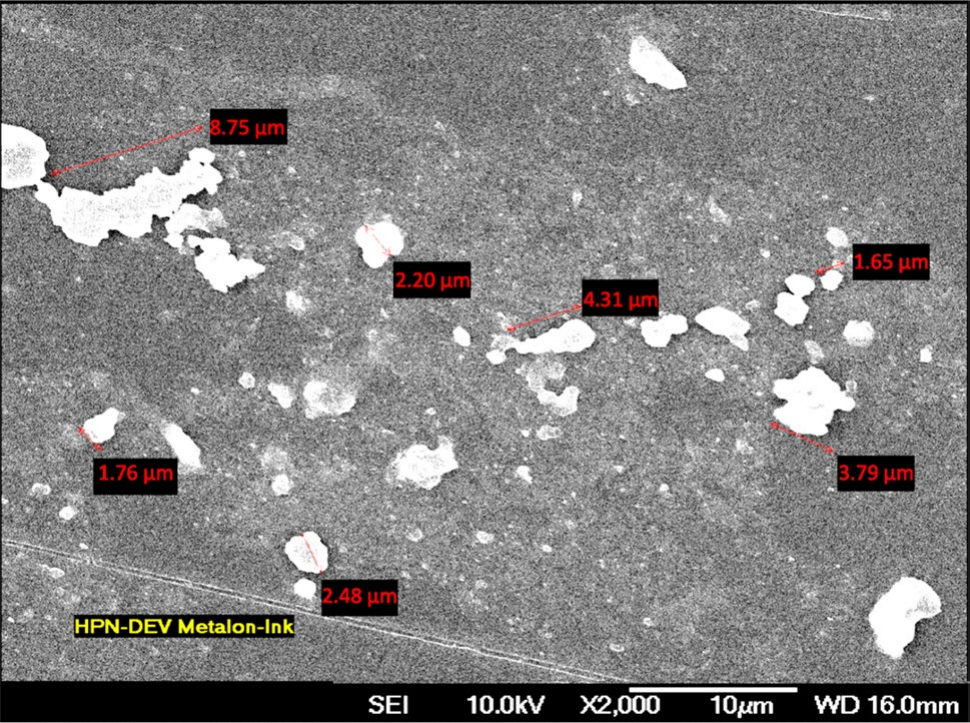
FE-SEM images of Ni flake particles at 2000 × magnification.

During the screen printing process, ink paste is exposed to various regimes of shear forces, while the squeegee stroke pushes it through the screen mesh onto the substrate^4,14^. The low shear rate of 0.1 reciprocal seconds (1/s) corresponds to the ink paste at rest on the screen plate mesh before printing, while the high shear rates between 100 and 1000 1/s simulate the ink transfer assisted by the squeegee stroke^65^. At high shears, a several orders of magnitude drop in ink viscosity is expected for the ink to become more fluid to complete the ink transfer during printing^65^. Ni flake ink yields the expected low viscosity of 1000 Pa.s at high shear rates and a high viscosity of 1 Pa.s at low shear rates, as depicted in the steady-state rheology flow diagram in Fig. 2a; thus, ink can flow through the screen mesh to realize ink transfer. The FE-SEM images in Fig. 3 show that the majority of Ni particles averaged 3 µm ± 1, with a few outliers at 9 µm ± 4. The flake-shaped particles have no particular order in the suspension. When the shear force is applied, the flake particles start aligning in the direction of shear and exhibit less resistance to flow. When the shear is increased, the particles display typical shear-thinning flow, which is a distinct screen printing ink behavior^65^. To further understand the ink material, the rheology was measured at increased temperatures while the shear rate was kept constant at 100 s^−1^. Figure 2b shows that the viscosity not only decreases from 12 to below 4 Pa.s when the temperature is varied from 20 to 60 °C but also starts to experience a phase transition at approximately 35 °C. The shear thinning and phase change with increasing temperature suggest that Ni ink is a nematic liquid crystal material with a clear first-order phase transition behavior^69^.

**Figure 4:**
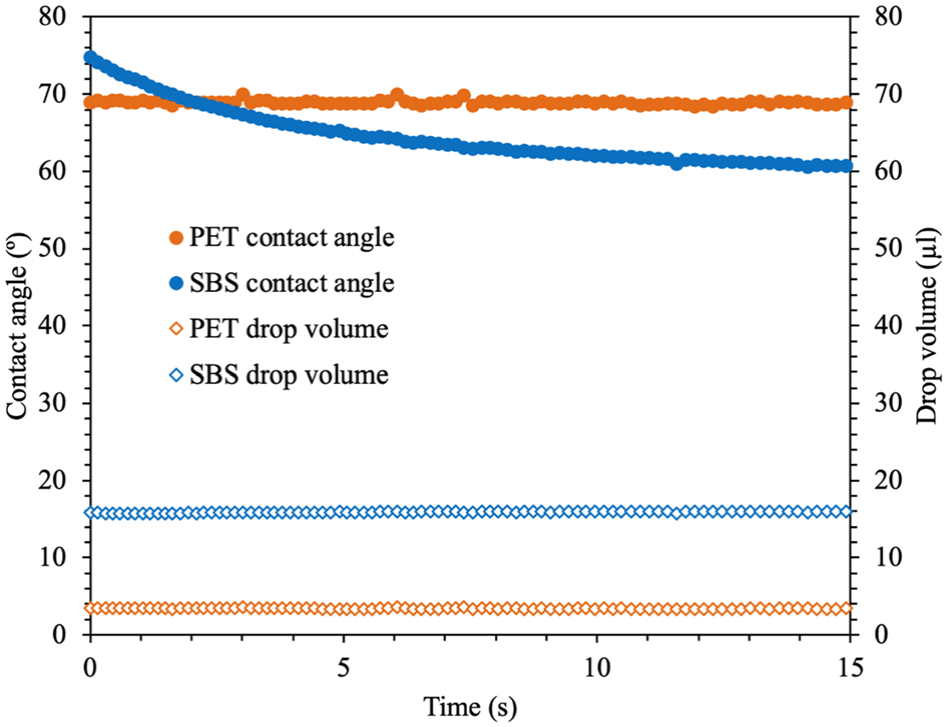
Contact angle of water on PET and SBS substrates over time.

**Figure 5:**
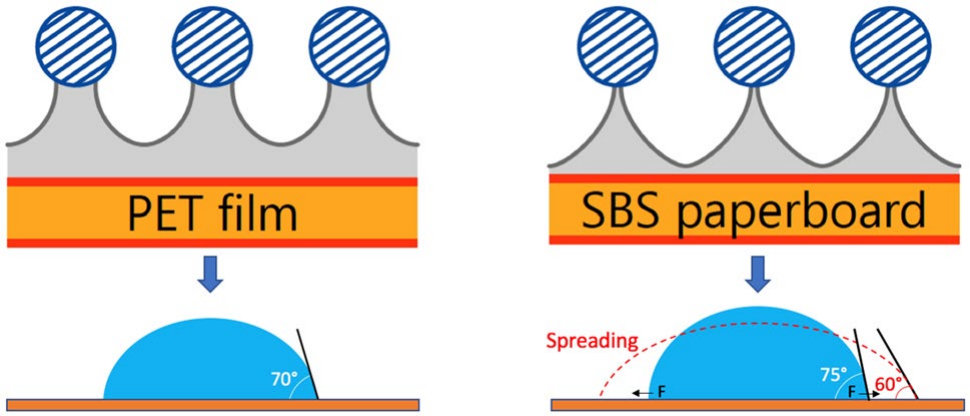
The liquid-bridge-type separation structure of ink release from the mesh during lifting of the tensioned mesh from the substrates: PET vs. SBS.

One of the main principles of homogeneous ink film formation on substrates is proper wettability^14^. The contact angle measurements presented in Fig. 4 reveal the wettability behavior of the substrates. The test liquid angle decreases when spreading on porous SBS, while it stays stable on nonporous PET over time. The liquid volume data confirm the decrease in the angle on SBS due to spreading since the liquid volume stays stable over time. When inks are applied on porous substrates, the soluble chemical compounds in the ink formulation, called vehicles, dissolve in the coating during ink leveling and spreading^16^. The spreading and diffusion add more pressure to break the liquid-bridge-type structure that occurs during lifting of the tensioned mesh from the substrates in screen printing (Fig. 5)^70^. This would enable quicker ink release from the mesh, which leads to a decrease in the amount of ink usage for SBS.

**Figure 6:**
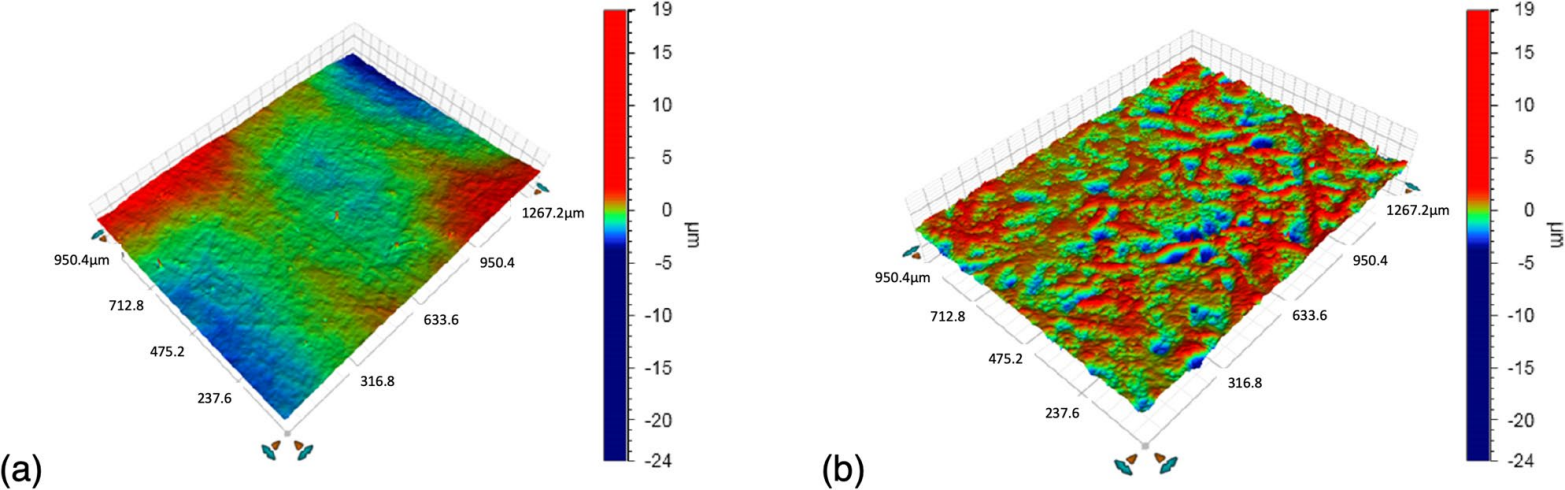
The profilometry surface images: (**a**) PET and (**b**) SBS.

**Figure 7:**
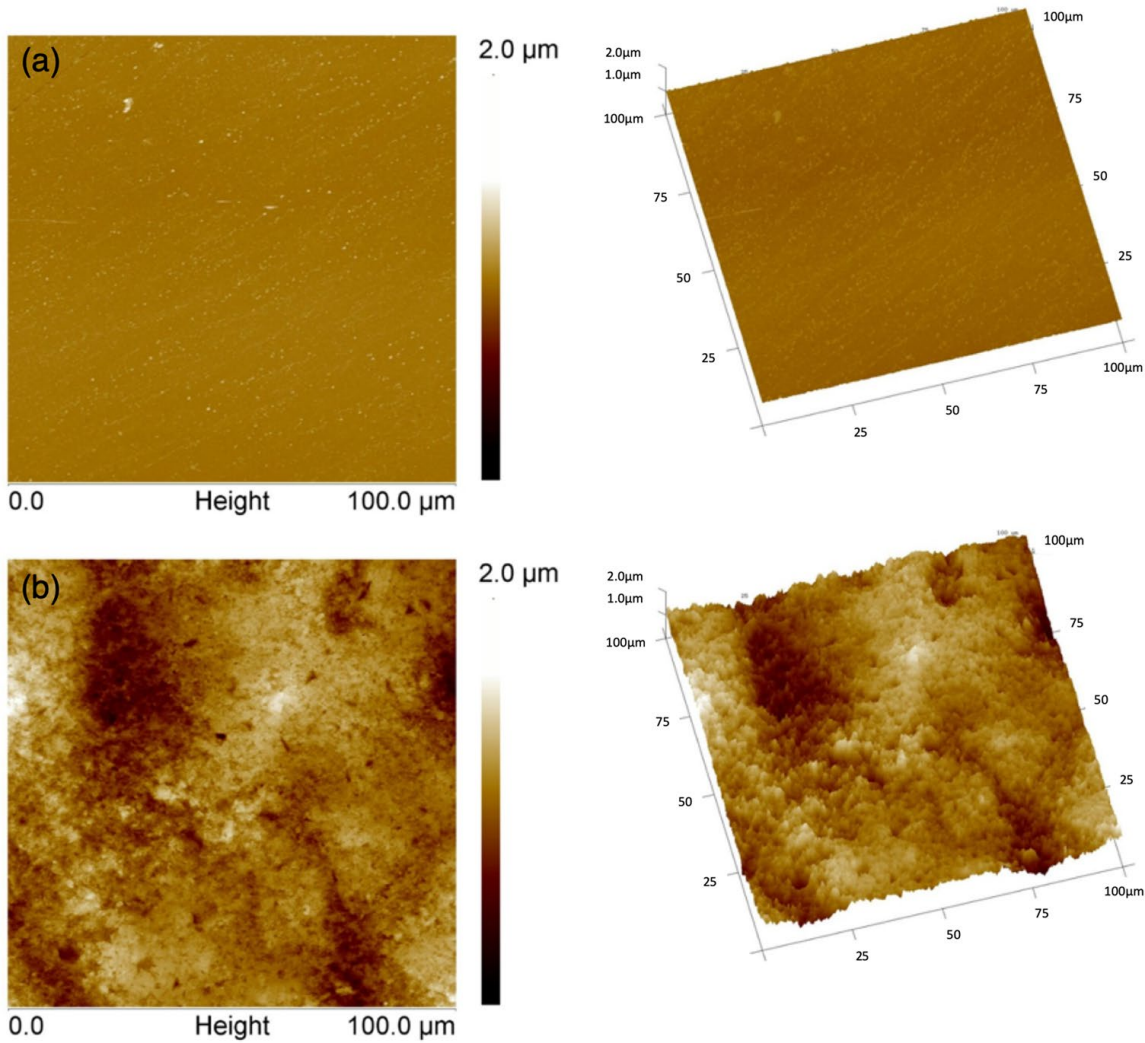
AFM images in contact mode: (**a**) PET and (**b**) SBS. Height images of 100 µm × 100 µm scan area. 2D (left) and 3D (right) views. Z-scale for the height images is 2.0 µm. Tilt = 30° and rotation = 15°.

**Figure 8:**
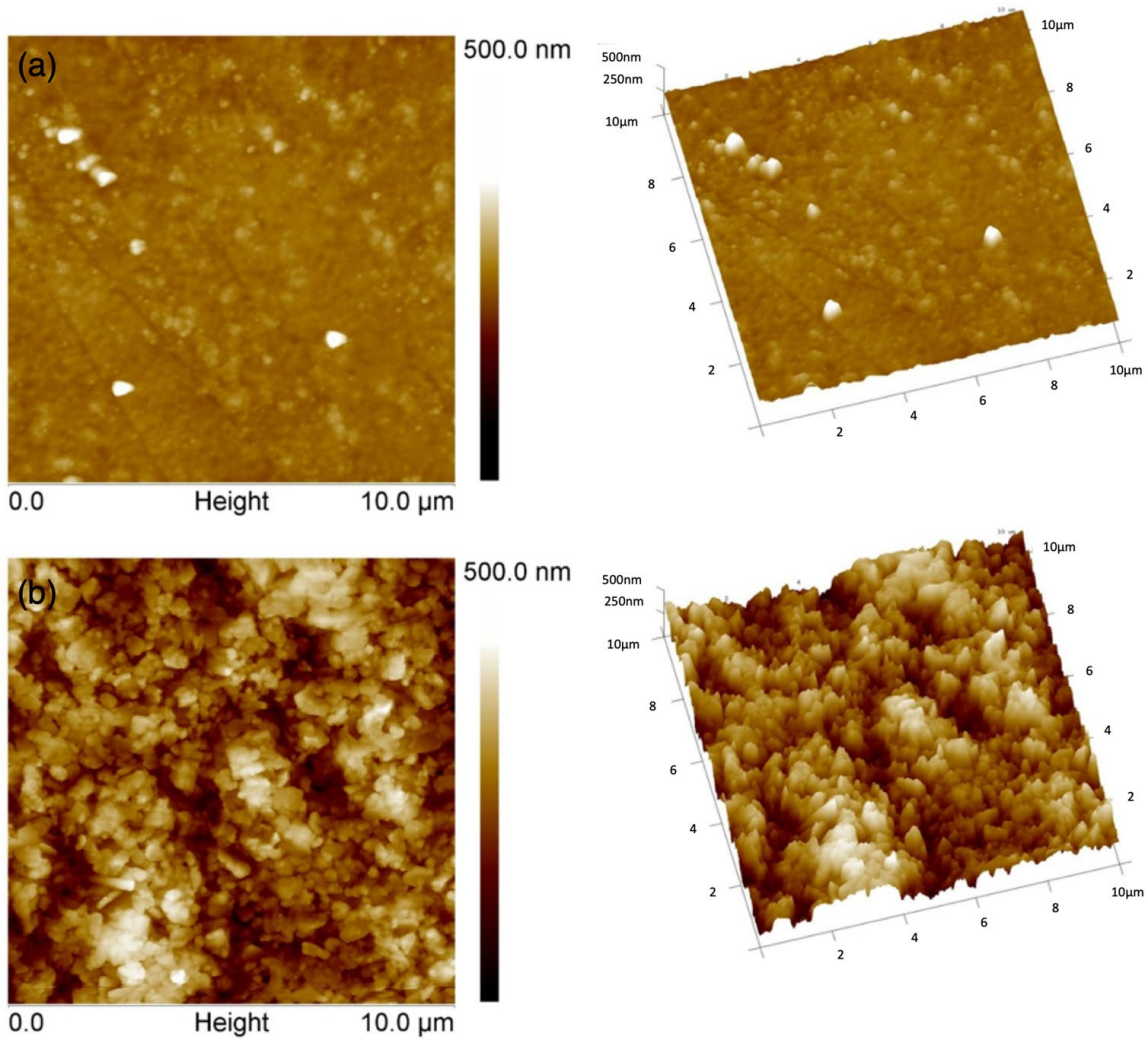
AFM images in tapping mode: (**a**) PET and (**b**) SBS. Height images of 10 µm × 10 µm scan area. 2D (left) and 3D (right) views. Z-scale for the height images is 500 nm. Tilt = 30° and rotation = 15°.

**Figure 9:**
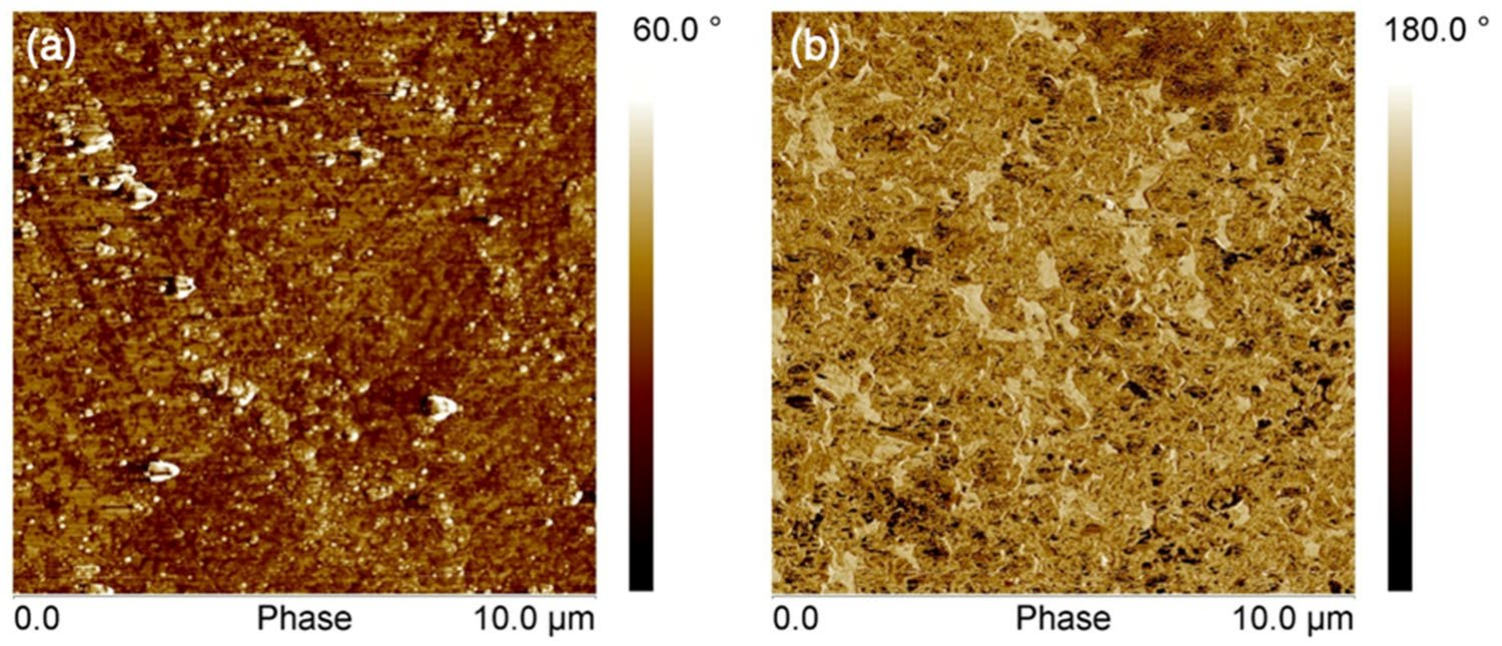
Tapping mode AFM phase images in 10 × 10 µm scan areas: (**a**) PET and (**b**) SBS.

Figure 6 shows 3D profilometry surface images of the substrates. Using this vertical scanning interferometry, the root mean square surface roughness is measured to be 17 nm ± 2 for PET and 1009 nm ± 10 for SBS, presenting a two orders of magnitude difference. In Figs. 7 and 8, AFM measurements both in contact and tapping mode also show that the PET surface is significantly smoother than the SBS surface. Bright particles in the phase images in Fig. 9 indicate the presence of filler material in the substrates. With AFM, the roughness is measured to be 12 nm ± 3 for PET and 176 nm ± 44 for SBS. The significant difference in the SBS roughness between the 3D profilometry and AFM methods is due to the length of the measurement scale of AFM^71^, while the interferometry scans depend on focus adjustment of the fringe contrast^72^.

**Figure 10:**
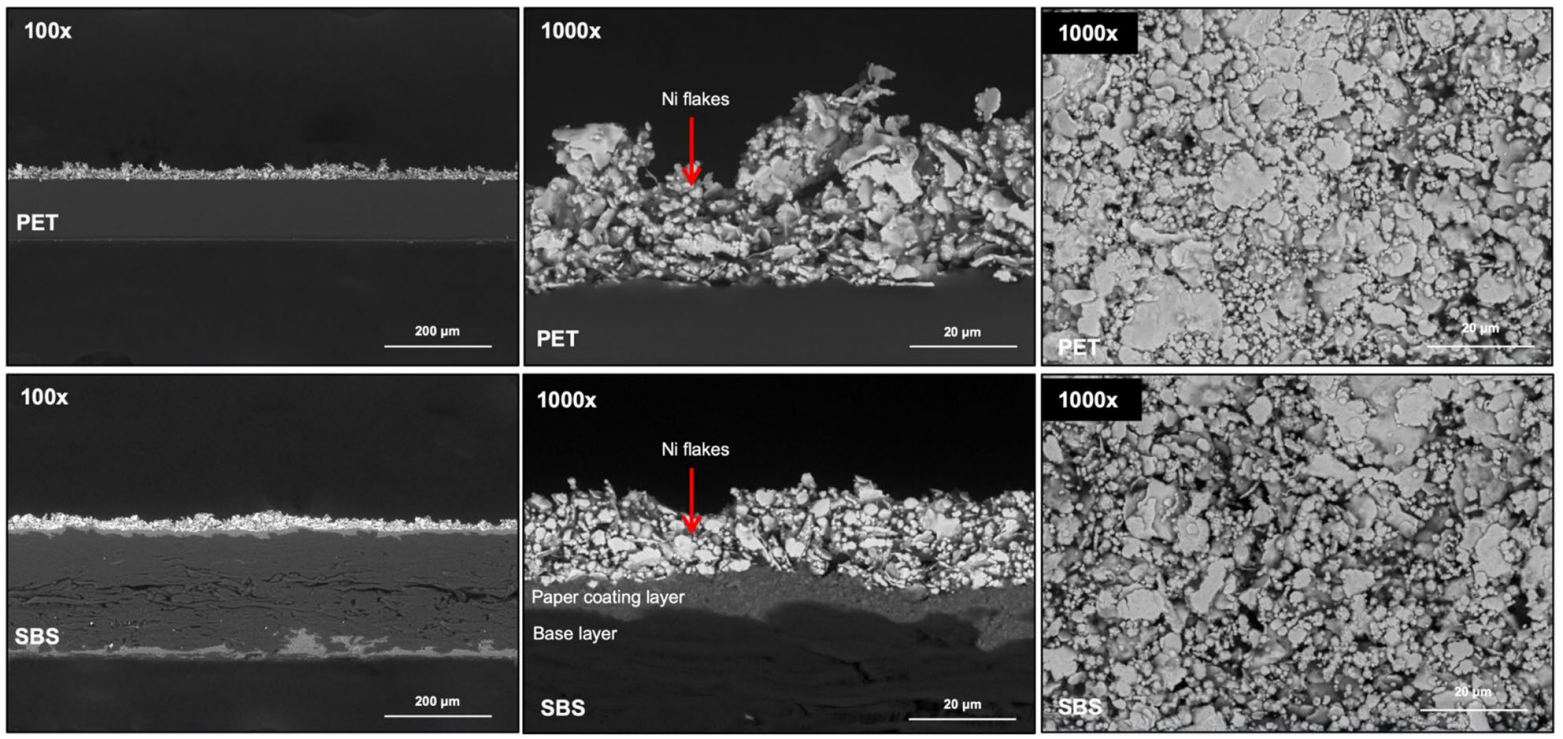
SEM microstructure images of the printed Ni ink at 100 × and 1000 × magnifications.

The SEM micrographs recorded from the printed samples at 100 × and 1000 × magnification are shown in Fig. 10 and reveal the microstructure of the Ni ink surface and the cross-sections. The images confirm the flake morphology of Ni particles as well as nonporous PET and porous SBS structures in the cross-section images. The coating layer on both sides of SBS that significantly improves the surface smoothness is also visible in the cross-section images. Using the digital gauge, the thicknesses of PET and SBS are measured to be 126 µm ± 1 and 252 µm ± 4, respectively. The higher standard deviation of SBS is attributed to a Poisson distribution, and the rough and porous paper microstructure containing open spaces, voids and pores is due to the cellulosic fibers and fillers^73^. The Ni ink film thickness is measured to be 52 µm ± 4 on PET and 36 µm ± 5 on SBS. The average ink weight is found to be 0.11 g ± 0.02 on PET and 0.09 g ± 0.01 on SBS, indicating that ~ 20% ink reduction is achieved by SBS. This result supports the previous findings that nonporous polymer substrates lead to higher ink usage than porous paper substrates^4,65^. A higher ink film thickness means more ink usage during production, which adds to the cost of electronic fabrication.

**Figure 11:**
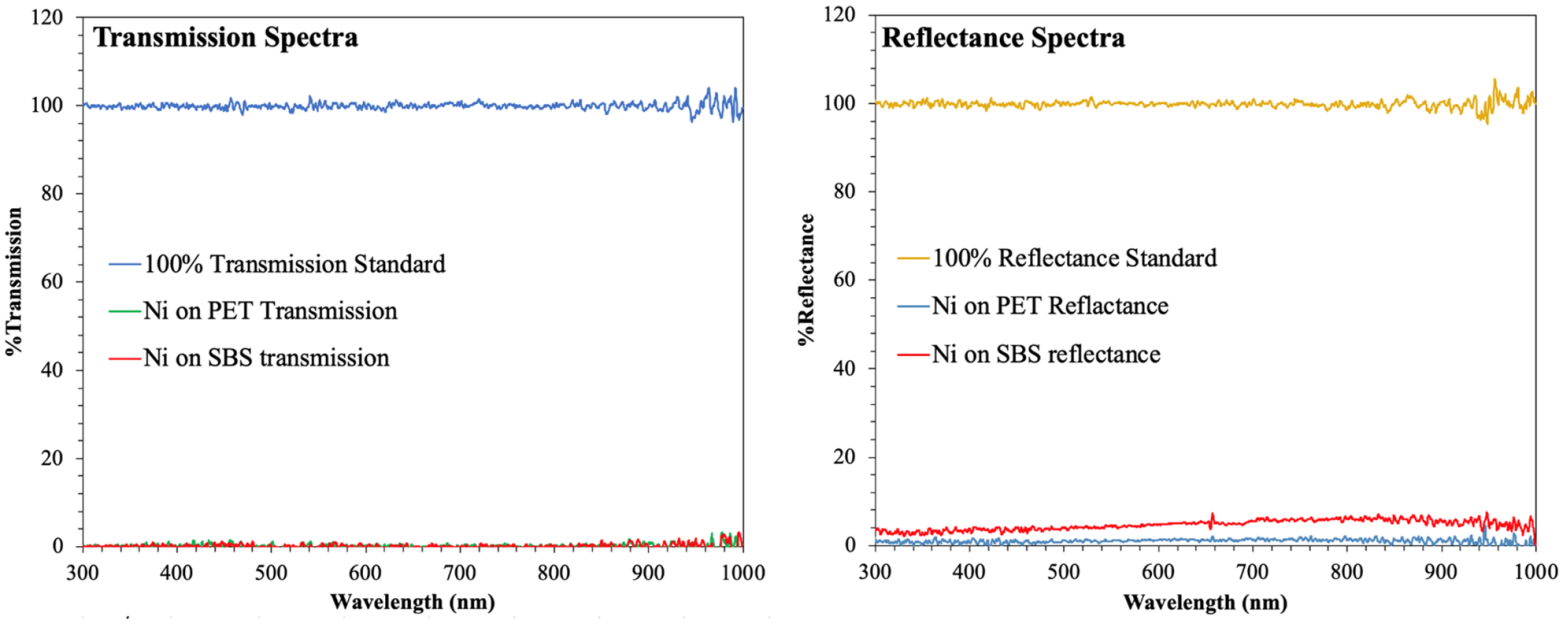
Optical property of printed Ni ink.

The working mechanism of photonic curing relies on the surface absorption of the printed Ni film heat-up and the thermal mass of substrates for cooling the heated printed ink layer by thermal conduction^22^. Figure 11 presents the optical transmission and reflectance spectra (300–1000 nm) of the printed Ni flake ink on both PET and SBS substrates. Photonic light emitted by the curing equipment also ranges from the mid-UV to near IR light region (300–1000 nm). Figure 11a demonstrates that the printed Ni layers are nearly 0% transparent over the defined spectral range on both PET and SBS substrates. The reflectance spectra of the printed Ni on both PET and SBS (Fig. 11b) demonstrate that approximately 3–5% of the incident light is reflected back in both cases, indicating that a vast majority (up to 97%) of the light was absorbed by the printed Ni.

Three conditions are reported for the optimum photonic curing of functional ink layers: x_f_ < x_s_, t_p_ < τ_s_, and τ_f_ < t_p,_ where x_f_ and x_s_ represent the ink film thickness and substrate thickness, τ_f_ and τ_s_ represent the thermal equilibration time of the ink film and substrate, and t_p_ represents a short pulse of intense pulsed light duration, respectively^22^. The thermal equilibration time of the materials is estimated using the formula in Eq. (1), where c_p_ is the specific heat (W s/kg K), ρ is the density (kg/m^3^), x is the ink thickness (µm) and K is the thermal conductivity (W/m K)^22^.

1 $$\tau =c\rho {x}^{2}/4K$$

**Figure 12:**
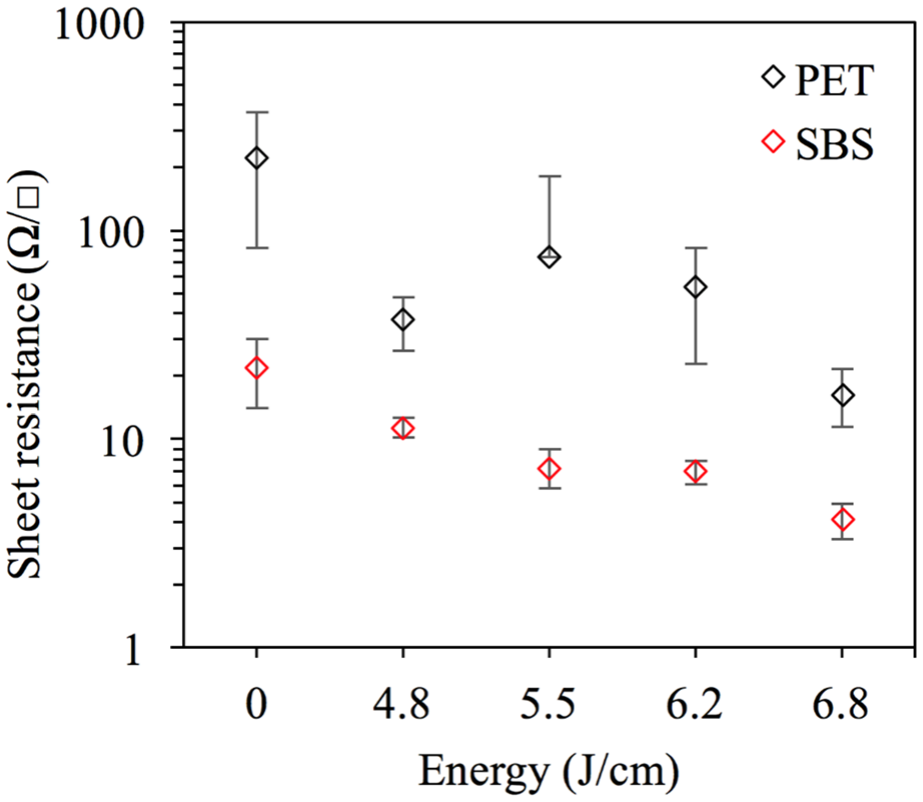
Sheet resistance of Ni ink on SBS paperboard and PET.

**Figure 13:**
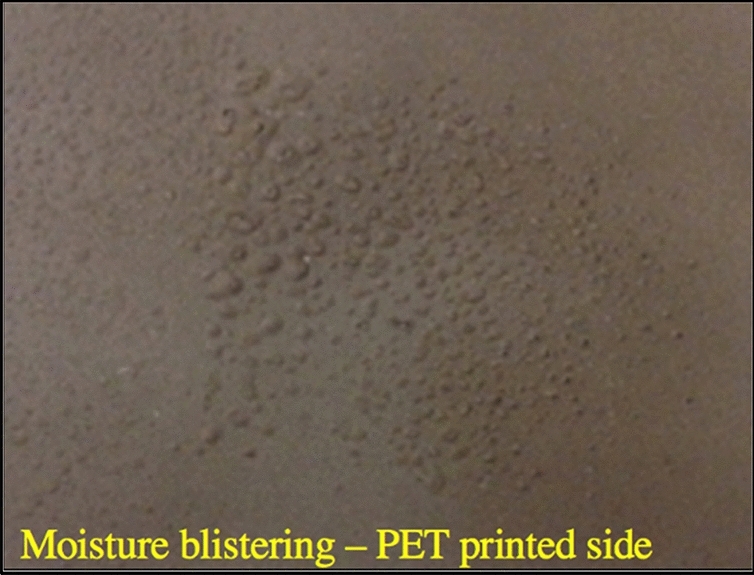
The blisters formed on the Ni ink film.

Tables 1 and 2 show the thermal equilibration time estimations and transient curing conditions, respectively, and the agreement between the thickness of the ink, substrates and peak processing temperatures satisfying the conditions for optimal photonic curing for all the experimental levels. After photonic curing, the lowest sheet resistance is found to be 4 Ω/sq on SBS and 16 Ω/sq on PET at a pulse length of 1.6 ms (6.8 J/cm^2^) (Fig. 12). The printed Ni resistance is found to be one order of magnitude lower than its noncured value and two orders of magnitude lower than the PET values. The results are comparable to those of conventional thermal heating at 130 °C for 5 min^65^. The Ni flake resistance values in this work are found to be one order of magnitude higher than the spherical particles of 5–500 nm on SBS and one to two orders of magnitude lower on SBS and PET, respectively, than the 50 nm particles (Table 3)^64^. The volume resistivity is calculated to be 1.4 × 10^–4^ Ωm for SBS and 8.3 × 10^–2^ Ωm for PET. The reason for the higher resistance and deviation observed with the PET sample is blistering formation caused by either moisture in the ink or partial vaporization due to the high working temperature (Fig. 13)^74^. The reduced surface quality and increased resistivity caused by blistering would be resolvable by applying a calendering process at higher nip to restore the quality and connectivity between the Ni particles^75^. The nip impression applies a high pressure in the z-direction such that the compression causes the functional material to experience plastic and viscoelastic deformations to induce alignments of the Ni particles in the ink layer and increase the contact between Ni particles. Thus, it improves surface quality by decreasing ink film roughness and increasing conductivity^75^.

**Table 1:** The simulation parameters and the estimations of thermal equilibration times.

Factors	$\mathrm{c}$(W s/kg K)	$\rho$(kg/m^3^)	$x$(µm)	$K$(W/m K)	$\tau =c\rho {x}^{2}/4K$(ms)
Ni on PET	440	8908	52	90.9	0.029
Ni on SBS	440	8908	36	90.9	0.014
PET	730	1370	125	0.24	16
SBS	1400	900	250	0.05	390

**Table 2:** Three transient curing conditions.

Factors^22^	Ni on PET	Ni on SBS
x_f_ < x_s_	52 µm < 125 µm	36 µm < 250 µm
t_p_ < $\tau$_s_	1 to 1.6 ms < 16 ms	1 to 1.6 ms < 39 ms
$\tau$_f_ < t_p_	0.029 ms < 1 to 1.6 ms	0.014 ms < 1 to 1.6 ms

**Table 3:** Comparison of Ni particle properties, sheet resistance and substrate type between the present work and other reported Ni films.

Metal	Curing	Particle shape	Particle size	Sheet resistance (Ω/sq)	Substrate	Author
Ni	Light irradiation	Spherical	5–500 nm	0.3	PI film	Park, Kim^64^
Ni	Light irradiation	Flake	3 µm	4	SBS paper	Present work
Ni	Thermal	Cubic	1–5 µm	5	PET film	Altay et al.^65^
Ni	Thermal	Cubic	1–5 µm	6	SBS paper	Altay et al.^65^
Ni	Light irradiation	Flake	3 µm	16	PET film	Present work
Ni	Light irradiation	Spherical	50 nm	500	PI film	Park, Kim^64^

**Figure 14:**
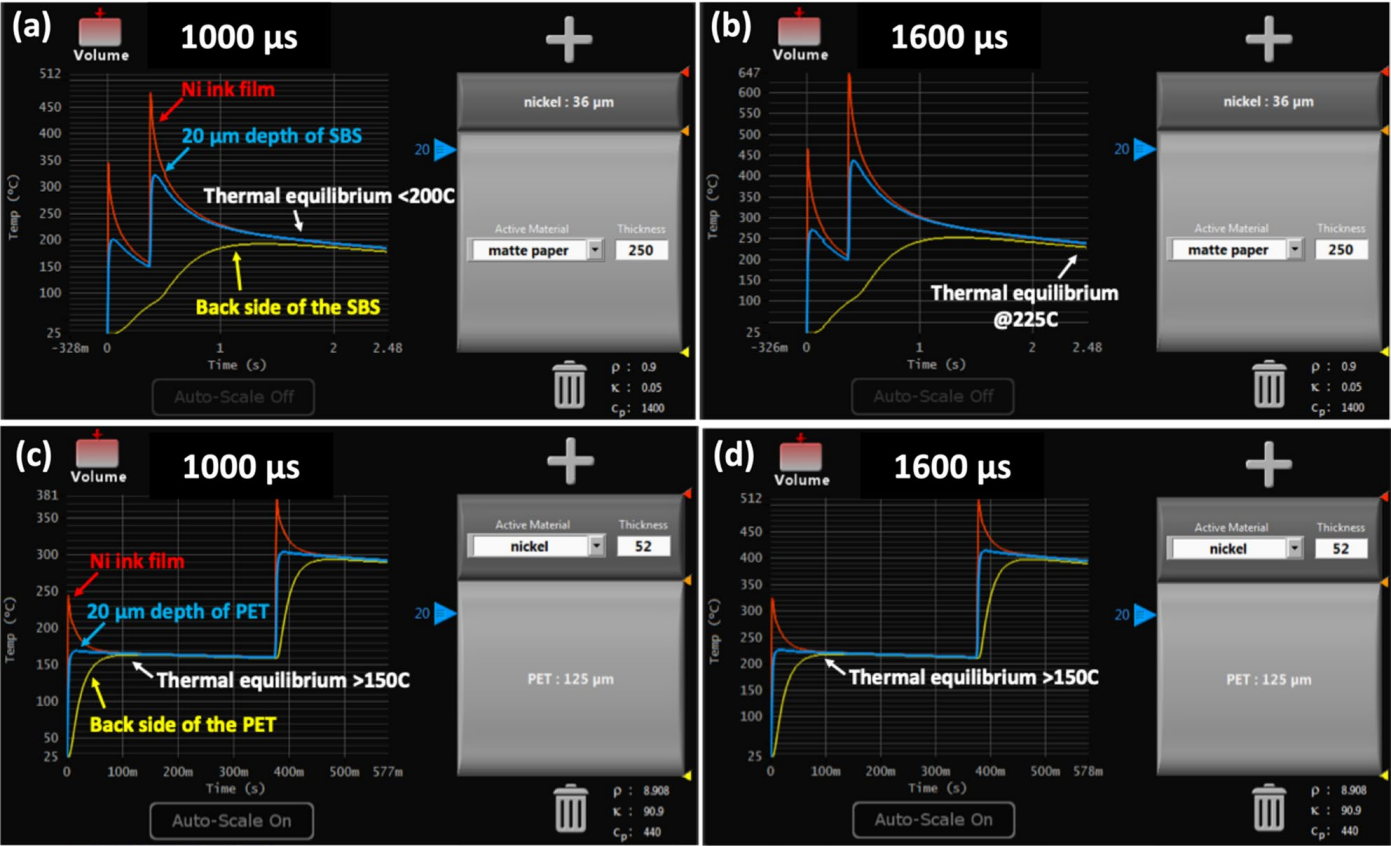
Simulation results by the SimPulse software: Thermal profile of Ni ink (**a**) at 1.0 ms on SBS, (**b**) at 1.6 ms on SBS, (**c**) at 1.0 ms on PET, (**d**) at 1.6 ms on PET.

The rapid high heating of the ink film occurs in such a short interval of time that the heat is effectively dissipated before reaching the back side of the substrate. When the temperature on the back of the substrate extends above a critical level at thermal equilibrium, such as the dimensional stability, ignition or shrinkage temperature, the integrity of the substrates may be lost. Figure 13 presents the simulation of the experimental sintering procedure using the SimPulse software. The data show that the light absorbed by Ni yields higher local temperatures of PET when light energy was applied at 1.0 ms (4.8 J/cm^2^) and 1.6 ms (6.8 J/cm^2^) pulse lengths, respectively. The heat generated on the ink film surface starts transferring to the PET substrate, and the temperature at the interface, 20 µm depth of PET (blue line), and back side of the substrate (green line) rise to a temperature below 250 °C where PET loses mechanical flexibility and starts to crack^41^. Thanks to the instantaneous nature of photonic curing, the time spent at the processed temperature is on the order of milliseconds, preventing an effect that would lead to a change in the physical properties of PET. In the case of SBS, the short pulse of light heats the surface of printed Ni ink to a temperature between ~ 500 and 650 °C. However, at the interface, the 20 µm depth of SBS only reaches 225 °C at equilibrium, which is below the ignition temperature (233 °C) and displays no deformation (Fig. 14). It must be noted that bulk Ni properties are employed during simulation. The properties of the Ni ink composite can be different from the pure material and lead to different peak temperatures during the process.

## Conclusions

In this work, we investigate the one-step photonic curing of a screen-printed Ni flake ink prototype on nonporous PET and porous SBS substrates for printed electronics applications. The flow rheology of Ni flake ink exhibits shear thinning behavior, which is the desired property for screen printing processing. The rheology at different temperatures revealed that the Ni ink prototype is a nematic liquid crystal. The characterization of the wettability behavior of substrates that controls the amount of ink transfer shows that ~ 20% less ink usage is achievable by using the porous substrate. Equivalent electrical performance is obtained from the Ni electrodes on both substrates despite the two orders of magnitude difference in surface roughness. The optimum sintering condition that provides the lowest resistance is achieved at a 1.6 ms pulse duration that corresponds to an energy of 6.8 J/cm^2^ and is comparable to the results gained from conventional thermal heating at 130 °C for 5 min. A minimum sheet resistance of 4 Ω/sq on SBS and 16 Ω/sq on PET is obtained, which is suitable for sensor applications.
